# Role of Vascular Smooth Muscle Cell Plasticity and Interactions in Vessel Wall Inflammation

**DOI:** 10.3389/fimmu.2020.599415

**Published:** 2020-11-26

**Authors:** Vitaly Sorokin, Keeran Vickneson, Theo Kofidis, Chin Cheng Woo, Xiao Yun Lin, Roger Foo, Catherine M. Shanahan

**Affiliations:** ^1^ Department of Surgery, Yong Loo Lin School of Medicine, National University of Singapore, Singapore, Singapore; ^2^ Department of Cardiac, Thoracic and Vascular Surgery, National University Hospital, National University Health System, Singapore, Singapore; ^3^ School of Medicine, University of Dundee, Dundee, United Kingdom; ^4^ Cardiovascular Research Institute, Yong Loo Lin School of Medicine, National University of Singapore, Singapore, Singapore; ^5^ Genome Institute of Singapore, A*STAR, Singapore, Singapore; ^6^ School of Cardiovascular Medicine and Sciences, James Black Centre, King’s College London, London, United Kingdom

**Keywords:** vascular smooth muscle cells, smooth muscle cell phenotype, immune-like, inflammation, atherosclerosis

## Abstract

The pathobiology of atherosclerotic disease requires further elucidation to discover new approaches to address its high morbidity and mortality. To date, over 17 million cardiovascular-related deaths have been reported annually, despite a multitude of surgical and nonsurgical interventions and advances in medical therapy. Existing strategies to prevent disease progression mainly focus on management of risk factors, such as hypercholesterolemia. Even with optimum current medical therapy, recurrent cardiovascular events are not uncommon in patients with atherosclerosis, and their incidence can reach 10–15% per year. Although treatments targeting inflammation are under investigation and continue to evolve, clinical breakthroughs are possible only if we deepen our understanding of vessel wall pathobiology. Vascular smooth muscle cells (VSMCs) are one of the most abundant cells in vessel walls and have emerged as key players in disease progression. New technologies, including *in situ* hybridization proximity ligation assays, *in vivo* cell fate tracing with the CreER^T2^-loxP system and single-cell sequencing technology with spatial resolution, broaden our understanding of the complex biology of these intriguing cells. Our knowledge of contractile and synthetic VSMC phenotype switching has expanded to include macrophage-like and even osteoblast-like VSMC phenotypes. An increasing body of data suggests that VSMCs have remarkable plasticity and play a key role in cell-to-cell crosstalk with endothelial cells and immune cells during the complex process of inflammation. These are cells that sense, interact with and influence the behavior of other cellular components of the vessel wall. It is now more obvious that VSMC plasticity and the ability to perform nonprofessional phagocytic functions are key phenomena maintaining the inflammatory state and senescent condition and actively interacting with different immune competent cells.

## Introduction

Cardiovascular disease is the leading cause of human morbidity and mortality worldwide, despite improvements and modern approaches to treat and intervene in this condition. Despite modern medical therapies with b-blocker, antiplatelet and hyperlipidemic drugs, thousands of patients continue to experience recurrent cardiovascular events (1). It is clear that further treatments in addition to rigorous lipid-lowering and antiplatelet therapy are needed to reduce the rate of cardiovascular events in this population. Improving our understanding of the molecular mechanisms underlying atherogenesis and disease progression remains a cornerstone to identifying novel therapeutic approaches (2).

Inflammatory mechanisms in the vessel wall have been extensively studied both *in vitro* and in animal models. Although inflammation is a well-accepted pathological mechanism in atherosclerosis, to date, it has not been translated to specific therapies applied in clinical practice. It remains unclear whether specific tailored immune-profile therapies represent a viable future direction for coronary artery disease management (3, 4). An update from the CANTOS trial revealed that interleukin (IL)-1B therapy, when applied in a heterogeneous population, reduced the rate of cardiovascular events in only 15% of the patients (5). Moreover, in the CIRT trial, treatment with a broad anti-inflammatory approach did not reduce cardiovascular event rates. Taken together, these findings emphasize that we must deepen our understanding of the inflammatory processes that occur in vessel walls and our knowledge of cardiovascular immunology to support the development of therapies that can be used in clinical practice.

**Figure 1:**
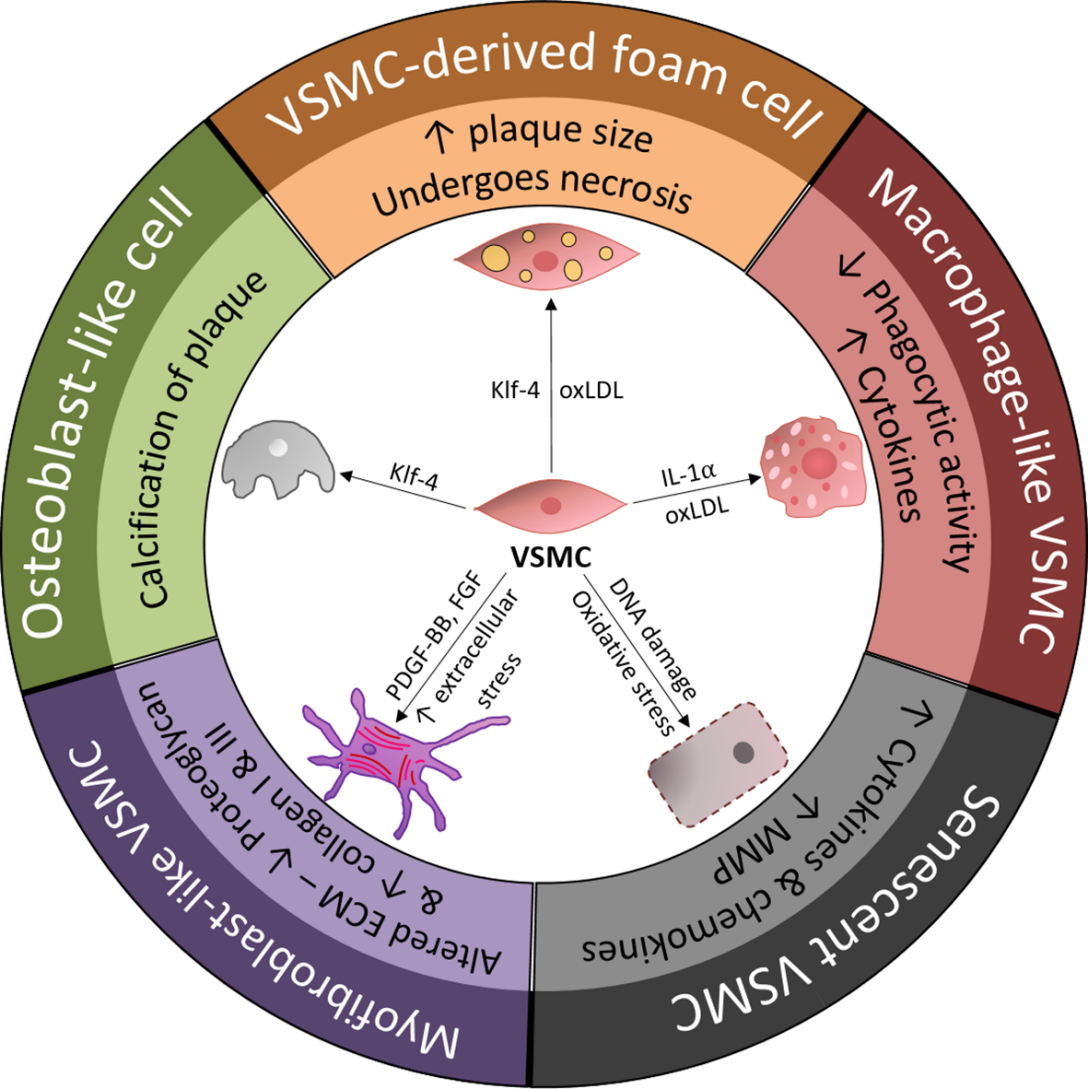
Overview of vascular smooth muscle cell (VSMC) phenotypic transition within the arterial wall and the main drivers of VSMC differentiation. ECM, extracellular matrix; FGF, fibroblast-growth factor; KLF-4, Kruppel-like factor 4; MMP, matrix metalloproteinase; oxLDL, oxidized low-density lipoprotein; PDGF-BB, platelet-derived growth factor-BB; VSMC, vascular smooth muscle cell.

Although inflammation in atherogenesis in humans is one of the initial steps following endothelial dysfunction and lipoprotein deposition, the most intriguing unresolved issue in this process is the lack of defined mechanisms initiating engagement of various immune cells (resident and nonresident) leading to the introductory secretion of pro-inflammatory mediators (6, 7). Endothelial cells (ECs) and resident myeloid-derived immune cells have been blamed for chemoattractive signaling affecting various immunocompetent cells and cytokine release (8, 9). It is now commonly accepted that endothelial dysfunction leads to the presentation of intercellular adhesion molecules (ICAMs, e.g., integrins and selectins), thus inducing immune-cell engagement and infiltration. However, extensive evidence has shown that the inflammatory process may critically depend on vascular smooth muscle cell (VSMC) plasticity and their ability to switch between different phenotypes (10, 11). Evidence has shown that VSMCs impact every step of atherosclerosis, and lineage-tracing studies have confirmed that atherosclerotic lesions are composed of at least 30% VSMC-derived cells (12). The intriguing ability of VSMCs to switch phenotypes and acquire properties relevant to different pathological states is complex (**Figure 1**). This plasticity of VSMCs is driven by biological stimuli from resident or nonresident cells in the vessel wall and is also strongly related to the proinflammatory molecular environment. An increasing number of studies have reported that VSMCs can develop characteristics reminiscent of fibroblasts, osteoblasts and even macrophage-like cells (12, 13). Recent investigations have indicated that VSMCs, as stromal cells, could express a less differentiated phenotype and subsequently dedifferentiate to a macrophage-like phenotype that plays an important role in vascular tissue homeostasis and efferocytosis (14). Studies have also shown that VSMCs not only acquire phagocytic properties but are strongly involved in cell-to-cell interactions with resident and nonresident immune cells. Intriguing data show that macrophage-like VSMCs acting as nonprofessional phagocytes and could lead to a chronic, nonresolving inflammatory state at the site of the vessel wall. This process initiates the migration of immune competent cells, including professional phagocytic cells and strong chemokine production (15). Macrophage-like VSMCs with ineffective phagocytosis could be related to an accelerated rate of programmed cell necrosis and an increased population of senescent and apoptotic cells (16, 17). Studies have demonstrated that apoptotic VSMCs and immune cells produce secretome rich in cytokines, including IL6, chemokine monocyte chemotactic protein-1 (CCL2) and ICAM-1 cytokines and adhesive molecules, that is highly inflammatory in nature (17).

Although the full picture of the association between atherosclerotic processes and inflammation has not been fully elucidated, the causal role of VSMCs and their plasticity in vascular inflammation is important. VSMCs with a macrophage-like phenotype that act as inefficient nonprofessional phagocytes need to be better understood and investigated to address the chronic proinflammatory state in atherosclerosis. Genetic studies, including fate mapping in animal models, provide new insights into existing paradigms, such as IL1β therapy (18). Advanced single-cell technologies and cell lineage tracking should further improve our understanding of these mechanisms (19).

## Identity of VSMCs in Diseased Vessels

One of the reasons that smooth muscle cell (SMC) identification and fate tracing is complex is related to the differences in the embryonic origin of VSMCs. For example, VSMCs in coronary arteries are derived from the epicardium, whereas those in the ascending aorta and arch vessels are derived from the neural crest, and those in the descending aorta are derived from somatic precursors. Hence, we must first identify the roles and phenotypic modulation of cells that share many phenotypic similarities but are also reported to display distinct genetic profiles and different responses to mediators, growth factors and pro-atherosclerotic stimuli (20). *In vitro* experimental work on the origin-dependent subtypes of VSMCs revealed that 3,604 genes are differentially expressed among them (21). Of interest, even though these subtypes were phenotypically and functionally indistinguishable, they responded differently to transforming growth factor-β1 (TGF-β1) stimuli, and the neural crest type of VSMCs showed autocrine production of TGF-β2 and platelet-derived growth factor subunit A (PDGFA), leading to their proliferation.

Historically, VSMCs within the vessel wall were identified by markers, including myosin heavy chain 11 (MYH11), calponin 2 (CNN2), smooth muscle 22α/taglin (SM22α/taglin) and smooth muscle cell actin (ACTA2). Advanced technologies tracing cell origin developed in recent years have raised questions about studies of VSMCs based only on marker identity (12, 22). For instance, VSMC-specific lineage tracing studies have revealed that previous results based on markers might have produced incorrect or inaccurate identification of VSMCs (12). This finding explains by fact that VSMCs in different environments can downregulate contractile markers such as SM22α, and upregulate another markers, including CD68 and ATP-binding cassette transporter 1 (ABCA1) (23). It is especially true in a proinflammatory state where disease tissue contains VSMCs in the different states of differentiation or dedifferentiation; hence, marker presentation could be difficult to interpret, disregarding technical issues of antibody specificity and sensitivity (24). Furthermore, myeloid-derived cells can induce the expression of VSMCs markers, such as SM22α and ACTA2. Although the specificity of different markers to identify VSMCs is questionable, MYH11 expression is relatively stable and may be accurate in identifying VSMC-originating cells regardless of phenotypic modulation. However, the identification of phenotypically-modulated VSMCs requires multiple markers. For example, MYH11+ cells that express CD68 may indicate modulated VSMC dedifferentiation to a macrophage-like phenotype (25). Another marker mentioned in the literature in relation to macrophage-like phenotype, such as the VSMC phenotype identified *in vitro*, is galectin-3 (LGALS3). An *in vivo* myocardin-deficient mouse model study used the marker lysosomal-associated membrane protein 2 (LAMP2) (MAC3) to trace macrophage-like VSMCs (3, 26). Kuro-o et al. showed that VSMCs involved in neointima formation in a rabbit model present embryonic smooth muscle (SMemb)/non-muscle myosin heavy chain (MHC) isoform B as a marker of a fibroblast-like, prosynthetic phenotype (27). Another *in vivo* study with a rat model revealed that cellular retinol binding protein-1 (CRBP-1) indicates VSMCs with a fibroblast-like phenotype (28). Advanced technology improved VSMC identification and led to a deeper understanding of the complex role of these cells in atherosclerotic progression, specifically in the process of inflammation. Developed epigenetic markers of VSMCs will help to identify these cells regardless of phenotypic modulation. An *in situ* hybridization proximity ligation assay could trace the VSMC-specific epigenetic code-identified histone modification (demethylation) at the *ACTA2* and *MYH11* gene loci using histological sections. The repertoire of histone demethylation marks at these loci is deemed restricted to the VSMC lineage (29). Moreover, histone dimethylation (H3K4me2) for *MYH11* is an epigenetic mark that can be traced and tracked to transdifferentiated VSMCs. *In vitro* studies showed that even after VSMC modulation of macrophage-like cells, this epigenetic marker is still present (30).

*In vivo* genetic cell fate mapping with the Cre-loxP system provides unique insight into the cell landscape in atherosclerotic lesions and helps to trace their origin. The Cre recombinase imprints a specific genetic event with marker protein expression, traceable by fluorescence marker. Cre-loxP has been a useful tool to trace cell lineages in the process of phenotypic switching or cellular dedifferentiation. Based on the chosen VSMC genes, Cre could target the *MYH11*, *ACTA2*, or *Taglin* promoter. Advances in technology have led to the development of genetically inducible animal models for fate mapping. In these inducible models, Cre recombination is constructed so that tamoxifen induction can activate the system *via* the T2 mutant estrogen receptor ligand binding domain ERT2 (CreER^T2^). The inducible fate mapping model allows the determination of the fate of targeted cells at a specific point in time by activating Cre-LoxP by tamoxifen. Smooth muscle-expressing *Cre*-lines reveal their high specificity and have led to tremendous progress in the field of VSMC discoveries. Cells expressing target genes of interest could be further studied at the tissue level or in culture, although in some studies, proliferation and growth of VSMCs from mutant mice was a concern (31). Experiments using MYH11 CreER^T2^-ROSA floxed STOP-eYFP apolipoprotein E (ApoE)^-/-^, for example, showed that up to 80% of cells traced with immunostaining alone could be misclassified (26). Although this system is robust, a few concerns with the system still exist. Expression of the targeted genes in visceral SMCs is one of them. For example, Cre ER^T2^-LoxP-targeted MYH11 gene expression could be detected in lung pericytes. Other potential problems are related to random sites of integration and differences in gene copies. Additionally, the method could have diverse sensitivity in specific floxed alleles (22). Another limitation, of course, is that the Cre-loxP system is designed for animals and cannot be used in humans at this stage.

Innovative *in vivo* single-cell technology provides an unbiased opportunity to decipher the cellular content of atherosclerotic tissue in a complex multicellular environment (32). The combination of single-cell next-generation sequencing and antibody-based immunostaining in the latest chromium single-cell profiling technology opens a new era in VSMC research (19). This system may detect genetic and epigenetic variations and segregation patterns of diverse genes and transcripts, emphasizing features of specific cells and their phenotypic modulation (32). However, mapping gene expression at the single-cell level lacks the spatial position of cells in vessel wall tissue. In a recent publication by Rodrigues et al., scientists developed new technology that allows the combination of single-cell RNA sequencing data and dimensional orientation of genetic imprint on single cells resolution. This so-called slide-seq technology transfers RNA data from tissue sections to the surface covered by DNA-coded beads with known positions, and hence is able to localize single cells and their spatial positions. Understanding cell dimensional position would better elucidate cell function and cell-to-cell interactions (33).

Altogether, the clear identification of VSMCs in the tissue, understanding their interaction and dimensional orientation are the key factors that allows the in-depth study of VSMC modulation and phenotypic switching. Although the regulation of VSMC phenotype switching has been studied and fundamental *in vivo* experiments are currently underway, it is clear that advance in single cells research would be very important to broaden our horizons.

## VSMC Phenotypic Modulation in Relation to Inflammation

**Figure 2:**
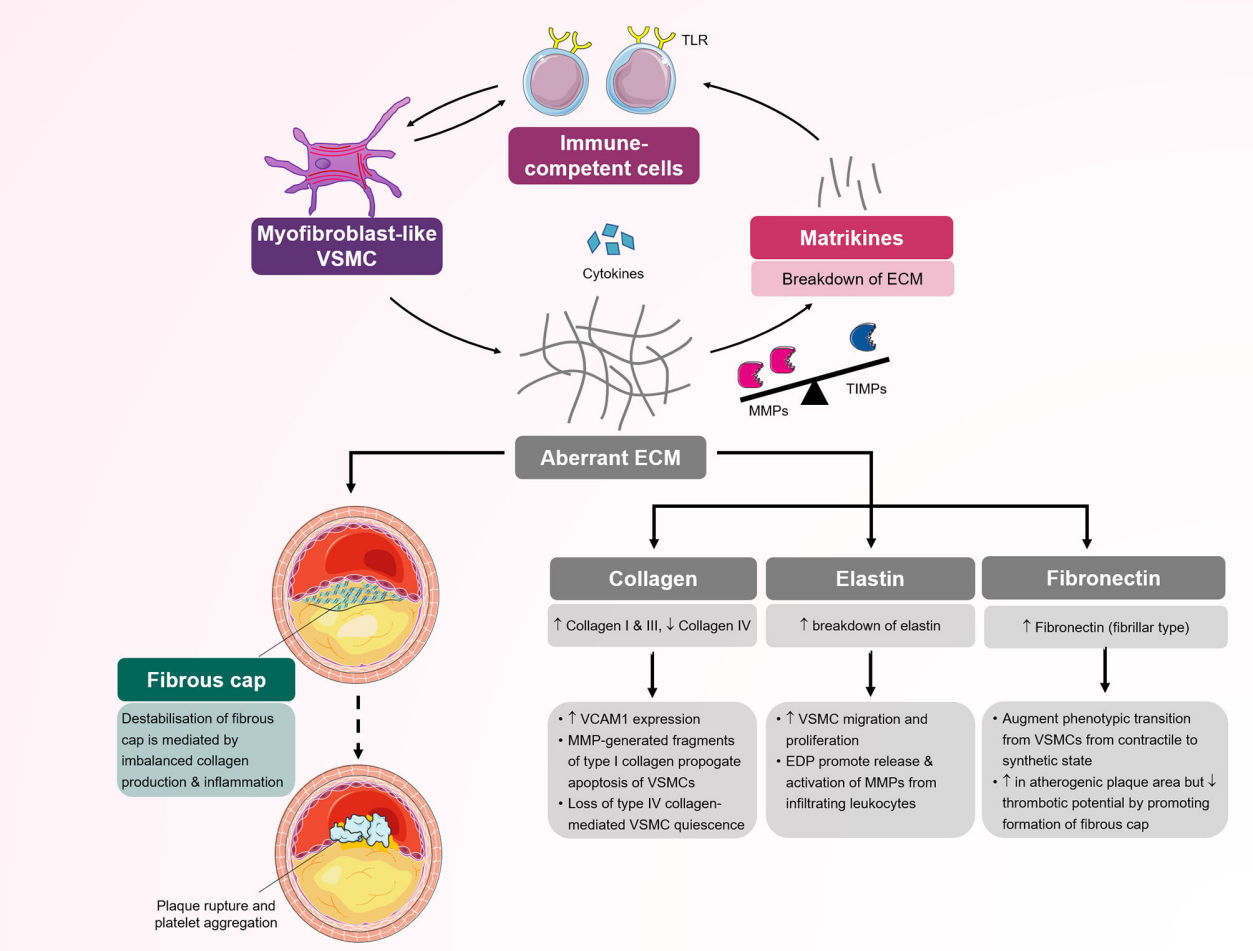
The roles of different vascular smooth muscle cell (VSMC) phenotypes in the initiation, progression and sequelae of atherosclerotic lesions. The normal artery has a trilaminar structure. The adventitia (outermost layer) contains vasovasrum, mesenchymal stem cells and collagen. The tunica media (middle layer) contains quiescent VSMCs, tissue resident macrophages and media progeny-derived VSMCs. Atherosclerosis begins in the intima (innermost layer). (1) VSMCs migrate into the tunica intima in response to inflammatory mediators and differentiate into macrophage-like VSMCs. Macrophage-like VSMCs and endothelial cells secrete chemokines, which attract circulating inflammatory cells, e.g. monocytes, which bind to adhesion molecules (ICAM-1, VCAM-1) before undergoing diapedesis and maturing into macrophages. Uptake of LDL by scavenger receptors leads to foam cell formation. (2) Low oscillatory shear stress, in combination with oxidative stress and hypoxia, promotes endothelial to mesenchymal transition (EndMT). (3) VSMCs contribute to the myofibroblast-like cell population, which promotes fibrous cap formation *via* the production of aberrant ECM. (4, 5) Mesenchymal stem cells (MSCs) originate from the adventitia, a source of VSMCs, and differentiate into mesenchymal-derived or osteoblast-like cells, which play key roles in plaque and vessel calcification. (6) In late atherosclerosis, macrophage-like VSMCs can negatively influence atherosclerotic plaques by increasing the expression of MMPs, cytokines and neutrophil recruitment. (7) Senescent VSMCs located in atherosclerotic plaques exhibit susceptibility to apoptosis or undergo necroptosis, which contributes to the generation of a pro-inflammatory environment (e.g., the production of cytokines (IL-1, IL-6), MMPs and chemokines). ECM, extracellular matrix; IL, interleukin; LDL, low-density lipoprotein; MMP, matrix metalloproteinase; TLR-4, Toll-like receptor-4; TNF-β, tumor necrosis factor-β; VSMC, vascular smooth muscle cell.

The process of VSMC engagement in phenotypic modulation is a matter of debate. VSMCs, as stromal cells of the arterial wall, phylogenetically develop in parallel with the pressurized vascular system (34). Under a physiological state, VSMCs as part of the media layer are exposed to overt mechanical or biological stimuli of circulating blood. It is now clear that VSMCs are directly or indirectly (via cell-to-cell or cell-to-ECM interactions) capable of sensing environmental changes, such as mechanical stress, hyperlipidemia and even acute myocardial infarction events (35). For example, tensile wall stress could be directly sensed by VSMCs, and shear stress defines VSMC structure and function indirectly through ECs-VSMCs interaction (34, 36). Sensing environmental changes would lead VSMCs to acquire functional and phenotypical properties that address these impacts on the arterial wall. This process is known as phenotypic modulation. Although VSMC modulation is intensively studied in disease progression, its roots likely originate from the physiological response of VSMCs to changes in vessel wall homeostasis (37, 38). However, the acquisition of new functions by VSMCs in the process of phenotypic modulation might lead to a cascade of events promoting inflammatory processes and atherosclerotic disease (39, 40). In atherosclerotic lesions, VSMCs, with a primarily contractile phenotype, may acquire synthetic (fibroblast-like), osteoblast-like and macrophage-like phenotypes (**Figure 2**).

In phenotypic modulation, the decrease in contractile properties is the most studied phenomenon. This crucial step is influenced by different chemokines and heavily regulated *via* contractile transcripts*. In vitro* cell culture studies clearly showed the response of VSMCs to mechanical stress leading to proliferation and synthetic to contractile phenotype modulation (41). Conversely, VSMCs cultured *in vitro* lose their myofilament structures and produce prominent Golgi complexes, indicating enhanced synthetic function. Along with the diminished contractile function, cells present the ability to produce a variety of mitogens with platelet-derived growth factor (PDGF) and basic fibroblast growth factor (bFGF) (42). PDGF with two B subunits (PDGF-BB) plays an especially important role in phenotypic VSMC modulation. In an experimental atherosclerotic model of SM22α*GC*–β-gal/ApoE^−/−^ mice, PDGF-BB promoted the expression of Sp1 protein, and this protein repressed the SM22α and MYH11 promoters *via* the G/C *cis-*element, hence downregulating the contractile phenotype and leading to phenotypic modulation (43). Phenotypic switching is known to be a multifactorial regulatory process in which myocardin has an important role. Ackers et al. used myocardin (Myocd)^+/-^ ApoE^-/-^ mice to show that inflammatory processes and macrophage-like phenotype activation were accelerated in myocardin-deficient mice (3). Interestingly, this study demonstrated that the myocardin-deficient state in heterozygous Myocd^+/-^ accelerated the atherosclerotic process *in vivo*.

Another study describing the decrease in the contractile phenotype established that the regulatory mechanisms underlying phenotypic switching may be affected by hyperlipidemia and the accumulation of lipid components in the vessel wall, which are common phenomena in atherosclerosis (44). Using cultured VSMCs, Shankman et al. showed that an increased oxidized phospholipid load led to the activation of immune cell markers *via* a switching mechanism that appeared to be dependent on Kruppel-like factor 4 (KLF4). In this mechanism induced by oxidized low-density lipoprotein (oxLDL), the KLF4 factor suppresses the expression of VSMC genes (SM22α, ACTA2, MYH11) by binding to the G/C repressor element in the gene promoter and by inhibiting the binding of transcription factors to the CArG element mechanism, leading to phenotypic switching (26).

While VSMC transcript modulation in the process of contractile phenotype change was meticulously investigated, epigenetic regulation of this phenomenon requires further attention (30). A previous study used an *in situ* hybridization proximity ligation assay and found that in the *MYH11* locus, H3K4me2 is the epigenetic mark, which was retained in transdifferentiated VSMCs after phenotypic modulation. This work showed that even after modulation of macrophage-like cells, VSMCs would still present this epigenetic mark (30). Another study showed that dynamic histone modulation may be an important mechanism involved in phenotypic switches. Reactivation of the gene by conversion of 5-methylcysteine (5mC) to 5-hydroxymethylcysteine (5hmC) by ten eleven translocation enzymes is one such example (45).

The most intriguing and important phenotypic modulation in relation to inflammation is that in macrophage-like VSMCs. Macrophage-like VSMCs acquire characteristics reminiscent of immune cells but continue to express markers of the original VSMCs. VSMCs with a macrophage-like phenotype suppress the expression of classic VSMC markers, including SM22α/taglin and ACTA2, and turn on the expression of multiple macrophage markers, including CD68, CD11b and F4/80. Given the concurrent presence of cells in transition at different stages of phenotypic change, it is nearly impossible to identify these cell types based on CD markers alone. Modern lineage tracing technology has broadened our understanding of VSMC macrophage-like phenotypic modulation in atherosclerotic processes (46, 47). Based on genetically inducible fate mapping experiments, VSMC phenotype switching to a macrophage-like phenotype was confirmed *in vivo* model (39). The authors of this study used genetically pulse-labeled VSMCs with tamoxifen-dependent Cre- recombinase integrated into the endogenous SM22α gene locus (SM22-CreER^T2^) to show that VSMCs transdifferentiate under pathological conditions into a macrophage-like Mox phenotype and that these macrophage-like cells are present in atherosclerotic lesions (39). Again, this process decreased the contractile phenotype and acquisition of a macrophage-like phenotype, regulated *via* a master transcription factor that controls the contractile phenotype of VSMCs, myocardin, which in turn is regulated by miRNA 143/145 (48).

Macrophage-like VSMCs acquire properties of nonprofessional phagocytes and present various scavenger receptors including low density lipoprotein receptor-related protein 1 (LRP1), which enable them to influx low-density lipoprotein (LDL) (15, 49). This lipoprotein influx leads to foam cell formation. The 3D multicellular *in vitro* model used recently demonstrated the transformation of VSMCs into foam cells (50). *In vivo* work by Wang et al. with lineage tracing studies in Myh11-CreER^T2^, Rosa26^tdTomato/tdTomato^, ApoE^−/−^ mice in combination with chromatin precipitation confirmed that the majority of plaque foam cells are VSMC-derived (46). This study also demonstrated that the expression of ABCA1 was lower in foam cells originating from human VSMCs than in macrophage-derived foam cells. This observation emphasizes the diminished ability of VSMC macrophage-like cells to efflux or digest cholesterol and lipoproteins (46). Due to this limited phagocytic activity, these macrophage-like VSMCs loaded with esterified cholesterol promptly progress to foam cells and subsequently contribute to atherosclerotic plaque formation (51). The fact that macrophage-like VSMCs do not acquire the complete functional capacity of professional phagocytic immune cells to clear lipoprotein load and mediate efferocytosis leads to a chronic, nonresolving inflammatory status (14, 37, 48). Also, VSMCs with a macrophage-like phenotype involved in chronic inflammatory processes by producing different cytokines, IL1β, IL8, IL6, CCL2 and various adhesion molecules (52, 53). In turn, these cytokines further recruit immune cells and promote various processes. Even more importantly, a study showed that macrophages such as VSMCs, once they acquire a macrophage-like state, would produce inflammatory mitogens and cytokines on their own even without external stimuli and continue to drive pathological progression (42). Although cytokine involvement has been proven by multiple *in vitro* and *in vivo* investigations, studies focusing on different cytokine properties of inflammatory processes have produced controversial results. IL1β has been extensively studied and was originally consider a potential therapeutic target to attenuate atherosclerosis [4, 5]. To date, the CANTOS trial was not able to show efficacy of a human antibody against IL1β (canakinumab). A recent study by Gomez et al. using the *ApoE^−/−^ Myh11* Cre ER^T2^ R26R-YFP mouse model showed that IL1β has a more complex role in the atherosclerotic process than previously described. As this study showed, the effect of the same cytokine (IL1β) could depend on the stage of the disease. This study revealed that IL1β at the stage of advanced plaque development could promote atheroprotective changes *via* outward remodeling and maintain VSMCs in the fibrous cap (18).

The VSMC phenotype modulation of fibroblast-like cells is yet another documented phenomenon strongly related to many factors, including inflammation. VSMCs with a fibroblast-like phenotype contribute fibrous tissue to form the cap of the plaque, engaging in arterial wall fibrosis and neointima hyperplasia (54). The synthetic, fibroblast-like properties of VSMCs are driven by biological stimuli from resident or nonresident cells and biomechanical characteristics of the ECM surrounding VSMCs. Common pathophysiological mechanisms related to atherosclerosis contribute to modulating VSMCs to the fibroblast phenotype. Inflammation with cytokine production and hypoxia with elevated lactate act as a driver of VSMC synthetic properties (55). *In vitro*, IL-1β reportedly induces the VSMC proliferative state and affects ECM production by VSMCs with increased fibronectin expression and decreased production of laminin α4 but no change in collagen type IV production (56). Other cytokines actively affecting VSMC synthetic properties including tumor necrosis factor α (TNFα), IL-17, tumor necrosis factor β (TGFβ) and interferon gamma (IFNg) have been reported and widely discussed elsewhere (57).

**Figure 3:**
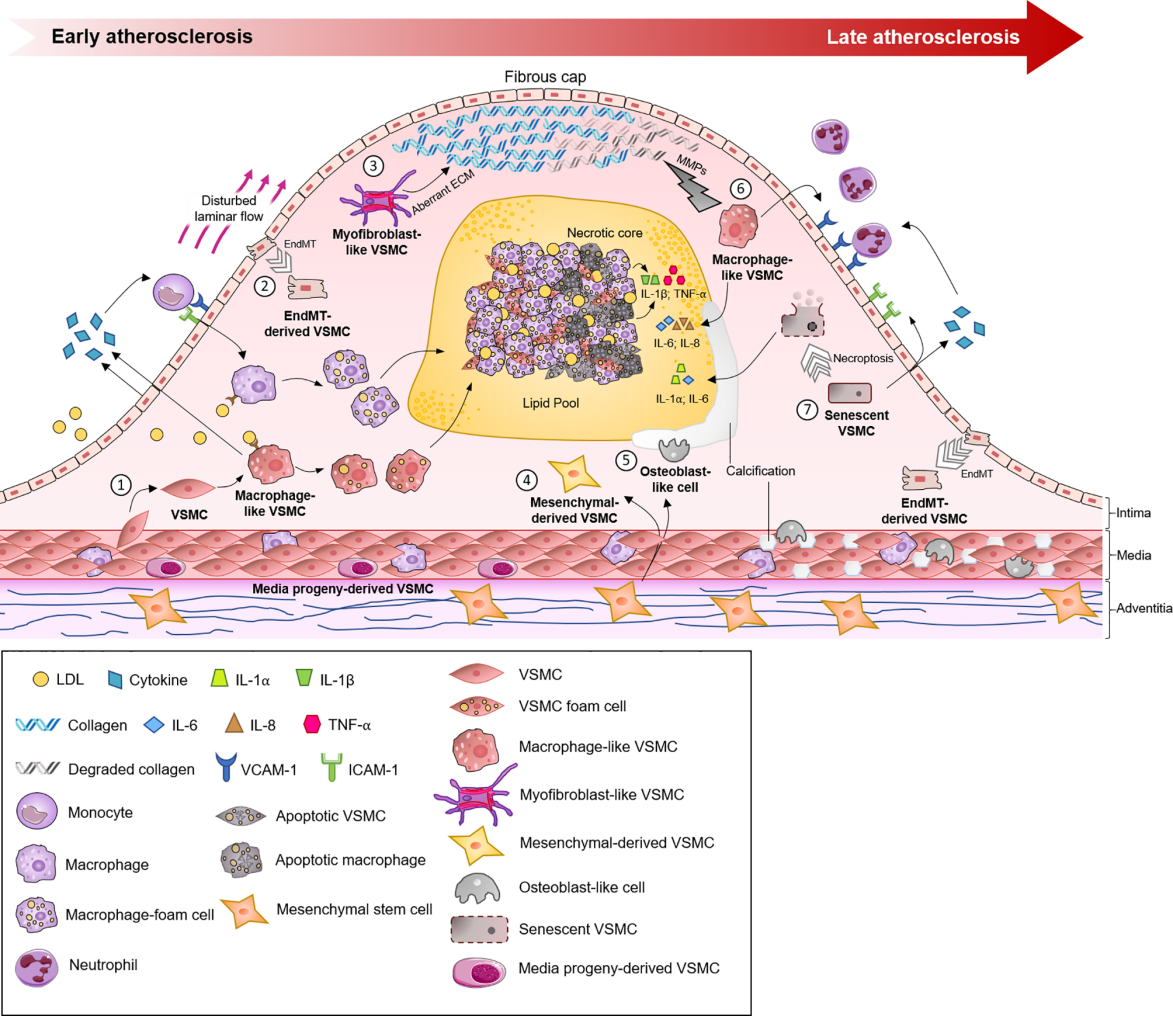
Schematic representation of several processes mediated by myofibroblast-like vascular smooth muscle cells (VSMCs) in atherosclerosis. Myofibroblast-like VSMCs are responsible for the production of extracellular matrix components (type I and II collagen and fibronectin) and inflammatory cytokines. Disequilibrium between MMPs/TIMPs results in selective ECM cleavage to expose bioactive molecular fragments and matrikines. Both matrikines and aberrant ECM further influence the inflammatory response by modulating chemotaxis and activation of circulating immune-competent cells. Infiltrating immune cells secrete cytokines, which further activate and promote phenotypic transition to myofibroblast-like VSMCs. Aberrant ECM deposition in the fibrous cap and activated inflammatory cells increase the risk of plaque fissuring and subsequent rupture. ECM, extracellular matrix; MMP, matrix metalloproteinase; TIMP, tissue inhibitor of metalloproteinase; TLR, Toll-like receptor; VSMC, vascular smooth muscle cell.

Although ECM is known to reinforce the wall structure, this is an oversimplified view of ECM function. Based on the available evidence, ECM is recognized as an active and highly dynamic provider of biochemical and mechanobiological signaling, that is involved in vessel wall immune modulation and could affect the inflammatory process (**Figure 3**). This view is true for modulated fibroblasts such as VSMCs. The properties of ECM produced by these fibroblast-like VSMCs may vary based on the status of the cell in its transition between the contractile and fibroblast-like phenotypes. Increasing evidence that suggests ECM molecules aberrantly produced by VSMCs can engage immune cells and promote inflammation. Cleaved by proteases, especially matrix metalloproteinase (MMP), the ECM provides fragments that function as biologically active peptides, called matrikines, which are involved in chemotaxis and cell activation (57). Collagen IV and laminin, which are produced by VSMCs with a fibroblast-like phenotype, are the main self-assembling components that form the basement membrane, one of the most important structures controlling cell movement in the vessel wall, including immune cell trafficking. Collagen I, collagen III and elastin contribute to more than 85% of arterial medial and adventitial ECM, and are also produced by modulated VSMCs (58).

The biological activity of ECM varies, but in general, it is accepted that collagen I, collagen III and fibronectin promote VSMC growth with elevation of extracellular signal-regulated kinase (ERK) phosphorylation and cell cycle regulators (59). Conversely, collagen IV stimulates myocardin expression and SRF binding to the promoter CArG box of the SM-MHC and SMα-actin genes, hence supporting contractile phenotype transition *in vitro* (60). *In vivo* laminin monomeric collagen I stimulate the production of inflammatory molecules such as vascular cell adhesion molecule (VCAM)-1, hence promoting the proinflammatory state. The same is true for collagen I, which increases VSMC apoptosis through calpain-mediated inactivation of anti-apoptotic X-linked inhibitor of apoptosis protein (xIAP) by increasing MMP1 production in human cells (61). Matrikines generated through cleavage of basement membrane molecules, including collagen IV and laminin α5, chemoattract immune competent cells. Collagen IV proteolytic fragments, particularly α2 with globular C-termini in the noncollagenase domain, inhibit angiogenesis. An *in vivo* study showed that vessel wall sites with low expression of the laminin α5β1βγ1 isoform on the basement membrane were used by neutrophils for preferential transmigration. Laminin also reportedly inhibits VSMC proliferation and prevents intimal hyperplasia (62, 63). Collagens, laminin and fibronectin interact with VSMCs *via* integrin receptors. Integrin receptors present a bidirectional signaling system with inside-out activation of extracellular ligands controlling binding function and outside-in activation where ligand binding to receptor mediates signal-to-cell function. The unique integrin outside-in signaling allows VSMCs to control the affinity to ECM containing RGD or LDV motifs and hence can regulate the effect of VSMCs and ECM interactions (59). Elastin, the most durable ECM with a half-life span of approximately 50 years, interacts with VSMCs *via* nonintegrin receptors. Elastin elicits an inhibitory effect on VSMC migration and proliferation, although the elastin-derived peptide, another matrikine, has the opposite effect on the vasculature. Elastin-derived peptide interacts with immune competent cells and promotes MMP1 activation, contributing to inflammation (64).

MMP production strongly correlates with ECM remodeling and the VSMC fibroblast-like synthetic phenotype (65). It is now clear that VSMCs with the fibroblast-like phenotype actively synthetize a spectrum of MMPs and their tissue inhibitors (TIMPs). The synthesis of 24 MMPs and 4 TIMPs in humans is transcriptionally regulated by several pro-inflammatory cytokines from resident and nonresident cells in the vessel wall (57). The theory that MMPs are the main functional molecules related to the cleavage of ECM based on *in vitro* data is challenged by an emerging “degradomic” concept from *in vivo* data that combines proteomic and genetic investigations (66). The MMP/TIMP enzyme system likely plays an ECM regulatory role in concert with cytokines, potentiating their effect, and selectively cleaving the ECM, which results in the formation of bioactive fragment matrikines.

VSMCs have also been implicated as integral players in intimal and medial vascular calcification, an adverse and highly regulated progressive process involving the osteoblast-like phenotype (67). This phenotypic regulation, characterized by upregulation of the bone-related transcription factors Runx2, Msx2, and Sox9, has been confirmed *in vitro* and *in vivo* (68). Of note, VSMCs that can dedifferentiate into osteoblast-like cells are not the only source of cells involved in the calcification process. Gli1+ mesenchymal stem cells (MSCs) can also differentiate into osteoblast-like cells and presumably therefore have an important role in the calcification process. Other studies have also demonstrated a role for circulating stem cells in vessel wall calcification (69, 70).

Vessel wall calcification begins as microcalcification in areas associated with the internal elastic lamina; patchy crystals form in these areas and later progress to calcified nodules that colocalize with atherosclerotic plaques. Although many trigger factors are involved in osteoblast phenotypic modulation, inflammation, cellular stress (mechanical or oxidative) and senescence are likely responsible (68, 71). However, microcalcifications themselves may trigger inflammatory processes, further accelerating calcification. The activation of NADPH oxidase and increase in hydrogen peroxide levels upregulate Runx2 expression, stimulating VSMCs to undergo osteoblast-like cell transformation. Inflammatory mediators in the vessel wall, such as tumor necrosis factor α, increase the expression of Msx2, which increases the expression of Runx2 and osterix (transcription factor SP7) *via* Wnt signaling (Wnt3a and Wnt7a). Runx2 binds to downstream genes to drive osteoblast-like transformation (67, 72). Furthermore, the upregulated transcription factor Runx2 has been shown to be responsible for osteoblast-like cell formation, whereas a decrease in myocardin levels is not (73). With diminished contractile proteins in the osteoblast-like phenotype, induced KLF4 binding to the promoter of contractile genes with repressed transcription occurs instead, as described earlier.

## VSMC to EC Interactions

EC dysfunction is well studied and extensively reported (8). The endothelium, beyond tone and vessel wall homeostasis, controls and heavily interacts with the underlying VSMCs. The effect of blood flow and shear stress on the vessel wall is a good contextual example. Lamellar blood flow and hypertension generate vessel wall shear stress, which affects ECs and leads ECs to undergo transcriptional modulation. If ECs are exposed to low levels of oscillatory stress and high levels of strain, they respond by increasing the expression of inflammatory molecules (74, 75). Inflammatory cytokines, such as IL-1β, TNF-α, and TGFβ, released by induced ECs affect the phenotype of the underlying VSMCs, induce the VSMC proliferative state, and promote synthetic and macrophage-like phenotypes. Increasing data have shown that in arterial walls, low oscillatory stress and high strain create imbalanced arterial wall homeostasis, which also leads to increased VSMC senescence and apoptosis (76). Moreover, ECs can generate the same proinflammatory response upon hypoxia, circulating immune stimuli, and oxLDL. In addition to the well-known LOX-1 expression, ECs present MHC II, acting as a conditional antigen presenting cell. A recent study showed that ECs act not only as “receptors” in the inflammatory response but also have the ability to enhance or suppress immune stimuli and deliver processed signals to the underlying VSMCs (8).

Communications between ECs and VSMCs take various routes. Direct connections *via* the adhesion molecules N-cadherin, VCAM1, and ICAM1 or receptor-ligand pairs such as EphB4 and ephrin B2 play important well-studied roles. The well-described interaction *via* small noncoding RNA functions similarly to secreted peptides and proteins. *In vitro* EC-derived miRNA-126, for example, increases VSMC proliferation, but miRNA-143/145 has an atheroprotective effect (9). Myoendothelial junctions structurally resemble the nerve synapse, which is another way to communicate between ECs and VSMCs, and is thought to depend on another adhesion molecule, connexin (especially connexin 40 and 43), and eNOS-derived nitric oxide (NO) production. Notably, NO production negatively regulates both PDGF isoforms: PDGF-AA and PDGF-BB. Experimental work on eNOS^-/-^ knockout mice revealed that negative regulation of PDFG by NO plays a role in VSMC proliferation and media remodeling. Additionally, interestingly, myoendothelial junctions represent one of a few mechanisms providing communication between VSMCs and ECs in bidirectional ways (77, 78).

Although the direct influence of ECs on VSMCs is well described, the effect of VSMCs on triggering EC dysfunction has not been fully elucidated. Based on *in vitro* studies, VSMCs cultured under endoplasmic reticulum stretch conditions produce macrovesicles that are able to affect ECs. Treating ECs with these macrovesicles induces endothelial dysfunction characterized by increased production of inflammatory markers (VCAM1, ICAM1, IL6 and IL1β) and drives EC apoptosis. C/EBP homologue protein (CHOP)^-/-^ knockout mice showed suppressed vessel wall infiltration of inflammatory cells and EC apoptosis (79). Transendothelial leukocyte diapedesis is another indirect mechanism through which ECs and ECM control the inflammatory process in the underlying vessel wall structures, including medial VSMCs. This heavily regulated process depends on EC-expressed adhesion molecules and intercellular junctional molecules (ICAM1, PECAM1, JAM, CD99), and the properties of the basement membrane also play important roles. The key proinflammatory state of ECs allows transendothelial leukocyte migration (67). Crawling leukocytes probe ECs and intercellular junctions with finger-like protrusions (“invasive protrusion”), which are enriched with actin and integrins. Recent studies using atomic force microscopy-enabled nanoindentation showed that protrusions sense the level of EC stiffness or weak EC actin density related to inflammation (80). Overall, despite progress, the full picture of transendothelial migration is under ongoing investigation. One of the few blind spots is related to the pericyte role in this process. This question needs more attention from the scientific community, as the current data are unclear.

## VSMC and Immune Cell Interactions

Atherosclerotic progression is driven by multiple phenomena in which inflammation plays a key role. In turn, inflammation is related to the innate and adaptive immune systems. EC activation and cytokine production by resident vessel wall cells chemoattract immune cells, which play crucial roles in disease propagation. Unfortunately, investigation of immune cell and VSMC cross-talk has been carried out mainly in mouse models, and translation of these data to humans, with questions about whether the mouse model faithfully mimics the human situation, is a matter of continuous debate (81). Another layer of complexity is added by the need to recognize the origin of cells expressing immune markers, as recent studies have revealed that a proportion of CD68^+^ cells involved in atherosclerotic lesion formation have a VSMC origin and immune cells share similar markers with VSMCs. This new evidence should be noted by scientists as previously published results are interpreted based on cell marker recognition approaches alone.

VSMCs, with their spectrum of different phenotypes extensively elaborated above, play different roles in the complex interaction with immune cells. The effect may vary through the different phenotypes, but in general, increased myocardin production in VSMCs inhibits leucocyte chemotaxis *in vitro* and attenuates vessel wall macrophage accumulation *in vivo* (3). Multiple studies have shown that modulated VSMCs (fibroblast- and macrophage-like phenotype) produce immune stimuli and express adhesion molecules, facilitating immune cell recruitment. In a study by Barlic et al., induced human coronary artery VSMCs upregulated CX3CL1 *via* the TNFα-NFkB signaling pathway. Expression of VCAM1 and ICAM1 by VSMCs in the atherosclerotic region, but not in healthy regions, has also been reported by other authors (82). Interestingly, both VSMCs with a macrophage phenotype and immune cells express a similar spectrum of inflammatory cytokines such as IL1β, IL8, IL6, and CCL2; hence, they may share similar functions. However, the physiological reason for VSMCs acquiring immune characteristics is not well understood. Whether VSMCs with macrophage-like phenotype function as “enhancers”, “modulators” or simply “effectors” of proinflammatory signaling generated by other immune competent cells or even by ECs needs to be investigated.

Leukocytes in the arterial wall are predominantly comprised of monocytes and T lymphocytes, but their origin and migration are under investigation and might be closely related to VSMC function and plasticity (83). Mononuclear cells clearly play a major role in vessel wall biology, which is dependent not only on the absolute numbers and subtypes of mononuclear cells but also on their activation status. It is helpful to divide monocytes in the vessel wall into the classic (proinflammatory), nonclassic (vascular maintenance) and intermediate subtypes. Generally, this classification is based on the expression (or absence) of CD14 and CD16. Recently, a study used mass cytometry to develop a more precise method to separate monocytes (84, 85). In that study, the authors noted that in addition to the well-known phenotype of classic monocytes, which includes high expression levels of CD14 and negative expression of CD16, proinflammatory monocytes also express CD36 and CCR2, whereas nonclassic monocytes express CD16 and CD11c and reduced human leukocyte antigen-DR isotype (HLA-DR).

Peripheral circulating mononuclear cells have been reported to be a source of monocytes that are recruited to vessel wall sites exhibiting inflammation (85, 86). A recent study showed that vessel walls are also populated by tissue-specific macrophages with origins distinct from those of peripheral blood-derived macrophages (PBDMs). Tissue resident macrophages embryologically originate from primitive macrophages directly, avoiding the fetal monocyte stage, as was clearly confirmed by human autosomal recessive mutation of interferon regulatory factor 8. This mutation leads to the depletion of circulating monocytes and spares tissue resident macrophages (87, 88). The origin of tissue-resident macrophages might be different in humans, although in mice, the yolk sac, fetal liver and bone marrow during the neonatal stage are sites. The embryonic origin of tissue-resident macrophages allows this long-lived macrophage subpopulation to self-proliferate and acquire tissue-specific characteristics (87). Studies involving transcriptional profiling analyses have demonstrated that macrophages presented outside the bloodstream have two distinct phenotypes: monocyte-derived macrophages that express CD206 and tissue-derived macrophages that lack CD206 expression (89). Studies have also shown that the roles of macrophages depend on their origin (90). PBDMs are heavily involved in inflammation and interactions with other immune competent cells. For example, PBDMs function in immunity by promoting the differentiation of FoxP3+ cells from naïve CD4+ cells (90). Tissue resident macrophages do not present proinflammatory properties, exhibit low MHC II expression and do not support T cell activation. The likely main function of tissue resident macrophages is related to homeostatic control in vessel walls with phagocytic activity to ingest apoptotic cells and debris (91). In addition to phagocytic activity, tissue resident macrophages interact with VSMCs to control arterial ECM, as shown in the experimental work of Lim et al. *in vivo* (92). Even so, for true tissue resident macrophages localized in the media, whether this pool of macrophages plays a functional role as so-called perivascular macrophages (PVMs) is unclear. PVMs are defined by Lappena et al. as macrophages located in contact or one cell thickness away from the abluminal surface of blood vessels. These cells appear to play a role in vessel permeability and are considered monocyte-derived macrophages. However, studies have shown that at least in the brain and heart, the PVM origin could be similar to that of tissue resident macrophages derived from the embryonic yolk sac (93, 94).

Macrophage polarization into the M1 and M2 phenotypes is relevant to the study of atherosclerosis investigations have shown that the M1/M2 ratio may indicate the status of the immune microenvironment (i.e., a proinflammatory or anti-inflammatory environment) in the vessel wall (95, 96). Traditionally, M1 macrophages are viewed as proinflammatory, whereas M2 macrophages are involved in chronic inflammation, healing and angiogenesis. M1 and M2 macrophages are conventionally distinguished on the basis of their expression of CD80 (expressed in M1 macrophages) or CD206 (expressed in M2 macrophages). The activation of macrophages and their polarization status may therefore indicate their engagement in immune processes. Polarization to M1 or M2 status can be initiated by different factors, including protein and nonprotein substrates. Recently, for example, a macrophage-activating mechanism involving the mediator MED1 was found to operate by transducing information from enhancers to promoters, thus leading to RNA polymerase recruitment (97, 98). The *in vivo* identification of polarized macrophages in lesions is another challenge that must be overcome to improve the understanding of immunological processes in the vessel wall. Efforts that used 18F-fluorodeoxyglucose positron emission tomography in combination with glucose uptake are inadequate for measuring macrophage activity and especially for distinguishing between M1 and M2 types. However, a recent study revealed that 2-deoxyglucose used in combination with glutamine accumulation might be helpful for distinguishing M1 and M2 macrophages in atherosclerotic lesions (99).

In the last decade, many cell types beyond monocytes have been reported to be active in relation to atherosclerosis. In particular, neutrophils, as part of the innate immune system, have been examined in mice and murine models, although neutrophil involvement in human atherosclerotic lesions is still an important question. CD66b is the most specific marker on human neutrophils, and data on human tissue reveal the presence of CD66b^+^ neutrophils in rupture-prone atherosclerotic plaques. Additionally, the analysis of CCL3 and CCL5 expression on human neutrophils revealed a correlation between their levels and mortality in patients with acute myocardial infarction (81, 100). Importantly, corroborating *in vitro* and *in vivo* experiments showed that inflammatory induction by PDGF-BB in VSMCs can attract neutrophils *via* the CCL7 chemokine. Neutrophils, once attracted to atherosclerotic plaques, promote VSMC death through the formation of neutrophil extracellular trap (NET) networks. In NETs, an extracellular neutrophil fiber network comprised of DNA and externalized histone H4 initiates lytic VSMC death and orchestrates plaque instability (101). The work of Silvestre-Roig et al. demonstrated direct contact of neutrophils and VSMCs in a hypercholesterolemic mouse model (101).

## VSMC Proliferation in the Inflammatory Process

The capacity of VSMCs to dedifferentiate and enter the proliferative cell cycle is supported by several studies *in vitro* and *in vivo*. However, the extent to which vessel wall inflammation influences VSMC proliferation or recruitment of various progenitor cells is a matter of ongoing debate (24, 102). A recent study using 10T1/2 cells as a model for VSMC differentiation, through CRISPR Cas9 deletion of CD146, showed that this protein plays an important role in VSMC transition from the proliferative to differentiated cell types. Using triple transgenic mice, this study identified a long-lived CD146^+^ VSMC lineage in the mouse aorta that was maintained at arterial branching sites in the quiescent state but entered the cell cycle and contributed to the proliferative pool of VSMCs in the vessel wall in the event of vessel injury (103). Similar findings of an extensively proliferative subset of VSMCs were reported by Dobnikar et al. The authors described the small population of VSMCs using Myh11-CreERt2/Rosa26-Confetti/ApoE^−/−^ mice. This differentiated subset of VSMCs demonstrated clonal expansion contributing to the pool of VSMCs with phenotypes identified in mouse atherosclerotic lesions. Another study with an experimental Myh11-CreERt2/Rosa26-Confetti mouse demonstrated by single-cell RNA sequencing that media contained a subset of stem cell antigen 1 (Sca1)-positive VSMCs showed the ability to downregulate the contractile phenotype and undergo phenotypic switching (104).

ECs in atherosclerosis have a broad program that enables these cells to initiate endothelial to mesenchymal transition (EndMT) (24). EndMT and its associations with atherosclerosis have been studied both *in vitro* and *in vivo*. *In vivo* data showed that the application of TGF-B promotes EndMT. Evrard et al. used the Cre-LoxP mouse *in vivo* model for lineage tracing and concluded that EndMT has an important role in atherosclerosis. In this process, EndMT-derived cells could differentiate into fibroblasts and smooth muscle-like cells (24, 105). Evidence from several studies clearly shows that EndMT is mediated by cytokines and strongly correlated with inflammatory conditions (106). However, EndMT was also strongly correlated with dyslipidemia, where Spillmann et al. revealed that EndMT was inhibited by high-density lipoproteins (107).

ECs are not the only source of smooth muscle-like cells in inflammatory disease. Adventitial MSCs also have important roles in vascular regeneration and atherosclerosis. MSCs in the vessel adventitia can be identified by their expression of the Gli1 marker in adult tissues (102). A study performed using the Gli1-CreER^t2^ R26td Tomato mouse revealed that a small proportion of Gli1^+^ cells isolated from microdissected mouse adventitia express the markers CD34, Sca1 and platelet-derived growth factor receptor β (PDGFRβ) and differentiate into VSMCs under specific conditions. Additionally, when exposed to PDGF-BB and TGF-β, these cells express VSMC markers, such as ACTA2, smoothelin and calponin (102, 108). These cells under pathological conditions could, therefore, form and be incorporated into the neointima of atherosclerotic lesions. Gli1^+^ cells can also be differentiated into osteoblast-like cells under other pathological and differentiation conditions and could potentially be involved in vessel wall calcification (102). Forthcoming experiments using cellular lineage tracing and single-cell RNA sequencing should reconfirm the involvement of adventitial progenitor cells in VSMC expansion. Recent cell fate mapping studies using Sca1-Cre ER CD45-Dre and PDGFRβ-LCL-Dre mice generated by CRISPR Cas9 revealed that PDGFRα+ but not PDGFRβ Sca1 VSMCs contribute to severe transmural injury of the vessel wall. These researchers also showed that the genetic ablation of Sca1+ cells or specific knockout of the Yap1 gene impairs vascular regeneration, thus identifying a putative role for adventitial PDGFRa+ Sca1+ progenitors in SMC accumulation and vascular lesion progression following severe transmural injury (109).

## Senescent VSMCs in the Inflammatory Process

An additional layer to the complexity of understanding VSMC biology includes cellular aging. Senescence is associated with cell death and the processing of cellular corpses. Senescence is accelerated with age and cell stress-induced damage. The entire process includes recognition of the senescence cell secretory phenotype by the innate immune system, followed by eradication of aging cells. Physiologically, senescent cells progress to apoptotic states, exposing on the surfaces “find me” and “eat me” receptors chemoattracting phagocytic cells, which they engulf as apoptotic bodies and dispose, avoiding activation of an inflammatory process. Tissue-resident macrophages are likely the cells that are involved in this dead corpse clearance in the physiological state. This process reduces the expression of proinflammatory signals such as TNFα and IL12 and increases the expression of anti-inflammatory cytokines such as TGFβ and IL10 (110). In atherosclerosis, VSMC premature senescence has been observed with diminished proliferative ability and increased cell death in atherosclerotic plaques. Increased senescence and apoptosis in atherosclerosis relates to lipid load, oxidative stress and other factors described in the literature (16, 17). These switches not only reduce the ability of VSMCs to maintain vessel wall ECM and stabilize structures in atherosclerotic plaques but also may overload the ability of tissue resident macrophages to maintain vessel wall homeostasis and enable PBDM recruitment (93).

In the atherosclerotic process, aging VSMCs present different senescent secretory phenotypes. Song et al. showed that aged VSMCs in a disease-prone mouse model exhibit a proinflammatory phenotype and overexpress IL6, CCL2, ICAM1 and Toll-like receptor 4 (TLR4) (111). Hence, given the proinflammatory phenotype of senescent VSMCs, it is likely that aged cells contribute to atherosclerotic progression. For example, in carotid artery atherosclerotic plaques, SaβG and the cyclin-dependent kinase inhibitors p16 and p21 are present, confirming senescent VSMCs with triple markers. Of interest, the presence of p16 characterizes replicative senescence, and p21 upregulation results from oxidative stress DNA-related senescence. The same study proved that telomeres of VSMCs in the atherosclerotic plaque were markedly shorter and barely detectable in advanced lesions (112). Additionally, other results showed that VSMC senescence contributes to the calcification of the vessel wall, although identification of the key mediators that accelerate this senescence process is underway and would be the next essential step (113).

Although cell senescence and apoptosis are naturally occurring processes leading to renovation of the cell pool in atherosclerotic plaques, exaggerated senescence and apoptotic inefficiency result in an increased rate of cell necrosis or so-called programmed necrosis-necroptosis. Necroptosis, as another form of programmed cell death, leads to cell lysis with exposure of cell debris and pathogen-associated molecular patterns or damage-associated molecular pattern responses to the extracellular environment. This form of cell death not only involves overexpression of the proinflammatory cytokines IL1, IL6, and IL12 but also leads to the uptake of cell debris by dendritic cells and subsequently presentation of antigens with activation of CD4+ and CD8+ cells with involvement of the adaptive immune system (17, 114). This process adds an autoimmune component to atherosclerosis with autoantibody formation to oxidized LDL or cardiolipin (115). Another reason for the inefficiency of apoptotic processes in atherosclerosis might be related to VSMCs with macrophage-like phenotype where phagocytic function is deficient. Studies by Clarke et al. using a conditional VSMC apoptosis SM22α-hDTR/ApoE^-/-^ mouse model revealed that VSMCs play a phagocytic role and express IL1α in atherosclerosis. In turn, expressed by macrophage-like VSMCs, IL1α induces other VSMCs to overexpress IL6 and MCP1 with perpetuation of the inflammatory process (16).

## Discussion

Overall, VSMCs are central players in vessel wall inflammation. A full understanding of the role of VSMCs in atherosclerotic disease, especially through inflammation, is yet to be comprehensively deciphered. In this issue, tracking VSMCs is key to studying their biology, and the results of studies that perform tracking based on a single or even a panel of markers may not always be accurate and have to be interpreted with caution. Hence, the application of advanced new *in vitro* and *in vivo* technology will be the key to progress.

To date, *in vivo* genetic studies using the Cre-LoxP system have been used to clarify the role VSMCs play, enabling precise cell lineage tracing, thus enhancing our knowledge of VSMC plasticity. The current trend to use inducible animal models supplemented with CreER^T2^ for fate mapping broadens scientists’ opportunities to trace the fate of targeted cells at a specific pathophysiological state. Currently pursued single cell resolution experimental work is another clearly a promising approach to identify the role of individual cells. For example, a recent single-cell RNA sequencing study has provided insight into the entire immunological landscape of the aortic wall and atherosclerotic plaque (52). The addition of proteomic information to data obtained using single-cell immune profiling and chromium single-cell immune profiling technologies promises to provide even more in-depth information about the cellular microenvironment and may also lead to a more sophisticated understanding, elucidating the reversibility of disease pathology in atherosclerosis (32).

Although new investigations with advanced methods would expand the frontiers of our understanding of VSMC biology, current data on the plasticity of these cells are intriguing. Multiple roles of these cells, fibroblast-like, osteoblast-like and macrophage-like, are accepted phenomena reported by multiple publications (116). However, the role of this plasticity in the contexts of inflammation and arterial disease is yet to be decoded. It makes sense to address this problem from the perspective that VSMC plasticity is likely related to constant hemodynamic stress and the vessel wall function of outward plasma lipoprotein convection (34, 38). Therefore, VSMCs, as stromal cells of the arterial wall not only response to wall tensile stress with contractile function and ECM production, in pathological circumstances would involve in clearance of plasma-borne molecules and even dying cells. These multiple tasks of VSMCs is clearly related to their phenotypic modulation and plasticity, although confines of the physiological and pathophysiological functions of VSMCs is the matter of debate in the literature. For example, Michel et al. considered the plasticity of VSMCs developed over hemodynamic evolution to maintain vessel wall homeostasis; however, this paradigm of stromal cell plasticity is involved in disease conditions (34). Lacolley et al. considered aging as one of the factors leading to loss of cell reprogramming, and affects cell plasticity and leads to cell senescence. Additionally, in agreement with Kolb et al. macrophage-like VSMCs with phagocytic properties are one example among other stromal cells that are involved in efferocytosis and other tissue debridement (38, 116). This point is especially important because the vessel wall media layer is poorly accessible by professional phagocytes, hence inefficient phagocytosis of VSMCs with macrophage-like phenotype might play a crucial role in the pathophysiology of atherosclerosis (37). Another question under discussion and ongoing investigation is very important to understand VSMC biology. Could every VSMC be involved in this phenotypic modulation process or do specific VSMCs undergo clonal expansion in the pathological state? According to Roostalu et al. the long-lived CD146^+^ VSMC lineage in the adult mouse aorta is maintained in a quiescent state, but enters the cell cycle and contributes to the clonal proliferation of VSMCs in the vessel wall in the event of vessel injury (103). A study by Chappell et al. clearly showed that clone cells are predisposed to any phenotypic modulation (117). In another study by Dobnickar et al. a subset of Sca1-positive VSMCs prompting to phenotypic transformation was identified in normal mouse aortae (104).

The effect of different phenotypic modulation of VSMCs on vessel wall inflammation and atherosclerosis is a matter of another discussion. Among other phenotypes, macrophage-like VSMCs are implicated in the inflammatory process. Macrophage-like VSMCs, as nonprofessional phagocytes, are reported to have limited ability to efflux and process plasma-borne lipoproteins. This nonprofessional phagocytosis could be sufficient in physiological circumstances but would be insufficient in hyperlipidemic state. The formation of foam cells as a result would generate a pro-inflammatory state, driving disease and attracting immune competent cells (40). Even though macrophage-like modulation is involved in disease propagation, Wirka et al., using a single-cell RNA sequencing method, identified modulated VSMCs in mouse and human atherosclerotic lesions that transform into fibroblast-like cells rather than a macrophage phenotype (52). These authors found that transcription factor 21 (TCF21) expression was strongly associated with fibroblast-like VSMC modulation in human disease. What makes this study more intriguing is the fact that fibroblast-like modulated VSMCs were associated with fewer cardiovascular events and might have a protective role.

In basic research, the ultimate goal is to identify a pathophysiological process that can be addressed in clinical practice. Vessel wall inflammation and VSMC plasticity are no exception. Sirolimus could be one example of the use of immunosuppressive effect of rapamycin to address VSMC proliferation and suppress restenosis. Drug-eluted stents utilizing this effect are commonly used to treat coronary artery disease. Another well-accepted therapy that influences VSMC remodeling and proliferation is angiotensin-converting enzyme inhibitors, which block angiotensin II production, hence changing VSMC proliferative and remodeling effects (118). Unfortunately, the situation with anti-inflammatory therapy is more complex and complicated by the absence of a clear understanding of inflammation in the context of arterial disease (36). Multitudes of reported chemo- and cytokines involved in inflammation often have multifactorial and codirectional effects. The cytokine IL1β could be a clear example of inflammatory process complexity. Although IL1β has been extensively studied and an antibody against this cytokine proved to be effective in attenuating atherosclerosis in a mouse model, the CANTOS trial showed as minimal as 15% reduction in the primary endpoint with an IL1β antibody (canakinumab) in a phase III clinical trial (4, 5, 119). Only recent study identified a reason for the low efficacy. The *ApoE^−/−^ Myh11* Cre ER^T2^ R26R-YFP mouse model shows that the IL1β effect might vary in different stages of the pathophysiological process and that in the advance stage of plaque development, it has an opposite atheroprotective effect (18). There are other examples that show promising results in murine models but reveal controversial or minimal anti-inflammatory effects in clinical trials (40). These results indicate again that, as murine models are far from human phylogenetically, human cells and tissue investigation using spatial single cell resolution is a very important strategy to understand vessel biology and develop appropriate anti-inflammatory therapies.

In conclusion, literature review clearly indicates that VSMCs are a key player in complex vessel biology. A recent investigation highlighted that different phenotypic modulation of VSMCs might promote or attenuate the inflammation. Deeper comprehension of VSMC plasticity would be possible using new technology to trace cell fate mapping or perform single cell studies. More investigations using human tissue on single cell resolution are needed to understand the clinically relevant VSMCs pathophysiology. The success of anti-inflammatory therapy, yet to be developed and impact clinical outcome, will strongly relate to advances in our understanding of multifactorial inflammatory processes in the human vasculature.

## Author Contributions

VS, RF, and CS conceived the ideas. VS, KV, and CW collected data and wrote the manuscript. VS, TK, XL, RF, and CS reviewed and edited the manuscript. All authors contributed to the article and approved the submitted version.

## Conflict of Interest

The authors declare that the research was conducted in the absence of any commercial or financial relationships that could be construed as a potential conflict of interest.
